# Health status in patients at risk of inherited arrhythmias and sudden unexpected death compared to the general population

**DOI:** 10.1186/1471-2350-11-27

**Published:** 2010-02-17

**Authors:** Anniken Hamang, Geir Egil Eide, Karin Nordin, Berit Rokne, Cathrine Bjorvatn, Nina Øyen

**Affiliations:** 1 Genetic Epidemiology Research Group, Department of Public Health and Primary Health Care, University of Bergen, Bergen, Norway; 2 Centre of Medical Genetics and Molecular Medicine, Haukeland University Hospital, Bergen, Norway; 3 Centre for Clinical Research, Haukeland University Hospital, Bergen, Norway; 4 Research Group on Lifestyle Epidemiology, Department of Public Health and Primary Health Care, University of Bergen, Bergen, Norway; 5 Department of Public Health and Primary Health Care, University of Bergen, Bergen, Norway; 6 Department of Public Health and Caring Sciences, Uppsala University, Uppsala, Sweden

## Abstract

**Background:**

The possibilities in the molecular genetics of long QT syndrome (LQTS) and hypertrophic cardiomyopathy (HCM) has made family screening, with diagnostic and predictive genetic testing part of the health care offer in genetic counselling of inherited arrhythmias, potentially affecting the subjective health among these individuals. The study compared health status among patients at risk of arrhythmia because of family history or clinical diagnosis of LQTS and HCM with reference health status scores of the general population.

**Methods:**

In the period 2005-2007, 127 patients (mean age 45 years, 53.5% women), with a family history of arrhythmia (n = 95) or a clinical diagnosis of LQTS (n = 12) or HCM (n = 19) referred for genetic counselling at the medical genetic departments in Norway filled in a questionnaire (Short Form Health Survey SF-36) measuring health status on eight domains. The patient SF-36 scores were compared to expected scores of the general population by t-test, and the relationship between the socio-demographic variables, clinical status, and SF-36 domains were analysed by multiple linear regression.

**Results:**

The total sample reported significant lower SF-36 score as compared to the general population scores for the domain of general health (mean difference -7.3 (<0.001). When analysing the sample in subgroups according to clinical status, the general health was still significant lower for the group of family risk and in the group of HCM. In addition the physical functioning, role physical, vitality and role emotional domains were reduced for the latter group. In general, employment, higher education and being referred to genetic counselling through a family member were associated with better scores on the health status domains.

**Conclusions:**

Having a genetic risk of arrhythmia affects general health significantly. In addition, patients with a clinical diagnosis of HCM demonstrate a significantly poorer health in both physical and mental domains.

## Background

Patients with long QT syndrome (LQTS) and hypertrophic cardiomyopathy (HCM) have a genetically based increased risk for serious arrhythmias. Both patients and family members therefore live with the threat of a premature sudden death. This threat applies especially to children and teenagers, but can also affect adults [1,2]. The advances in gene technology have made it possible to offer genetic testing, and at risk individuals and their close relatives have the opportunity to receive genetic counselling.

The penetrance (likelihood for actually developing the disease) is varying, which leads to uncertainty of ever experiencing clinical symptoms of the disease. The manifestations of symptoms can range from asymptomatic to sudden death, but there can be a lot of variation between individuals and families. The two diseases are structurally and functionally different from each other. LQTS is an ion channel disease leading to a prolonged QT interval with an increased propensity to ventricular tachycardia manifesting as torsade de pointes [3]. HCM is defined by the presence of increased ventricular wall thickness or mass, having ruled out hypertension and valve disease [4]. However, being genetic disorders causing arrhythmias they still have a lot in common in the genetic counselling setting, where topics like inheritance patterns, symptoms, management and prevention are being addressed according to guidelines [5].

Events and issues such as experiencing syncope, surviving life-threatening arrhythmias and living with an implantable cardioverter-defibrillator (ICD), have previously been shown to have an effect on health status [6-8]. It is also well documented that uncertainty about one's health can be very distressing [9,10], as well as the uncertainty regarding your children's health [11,12].

Living with the genetic risk of arrhythmia and possible sudden death may therefore affect health status of the patient group referred to genetic counselling. However, the knowledge of the health status of the patients coming to genetic counselling because of a risk of inherited arrhythmias is limited. Previous studies have mainly focused on children at genetic risk and their parents' higher distress levels as a reaction to that [13-15].

In the adult patient population a qualitative study of living with LQTS reported that these patients reported worries and limitations in daily life, but also here their main concern was for their children or grandchildren [16].

In adult HCM patients, living with HCM has been reported to be associated with decreased levels of self reported health status compared to the general population [17], and one study also reported problems for the patients to adjust to the diagnosis [18].

In Health research, the traditional outcomes mortality and morbidity have been supplemented with subjective accounts of health in patient groups of various diseases [19]. In general, health status is measured by instruments based on a multidimensional model of health, such as the Short Form Health Survey [SF-36] which is used in the present study. In SF-36, eight domains are constructed from the Medical Outcomes Study (MOS) (Boston, USA) which is relevant to functional status and well-being, and that can directly be affected by disease and treatment [20].

The patients coming to genetic counselling consist of high risk individuals with the potential to develop an arrhythmia. The arrhythmia risk may be present either because of a family history of arrhythmia or that individuals have a clinical diagnosis such as long QT syndrome. Similarly the HCM group consists of persons with a possible inherited risk not yet diagnosed and patients with the clinical condition.

Because there is limited systematic information about how the increased genetic risk affects the health status of the heart patients with LQTS or HCM, as well as their family members, we investigated the health status in a Norwegian sample of patients at risk of inherited arrhythmia prior to the genetic counselling session. Specifically, our research questions were; (1) Is there a relationship between living with genetic risk of inherited arrhythmia and health status vulnerability? (2) What is the relationship between socio-demographic variables, clinical status and health status domains among the patients at risk of arrhythmia coming to genetic counselling?

## Methods

### Participants

The participants comprised of (a) Norwegian patients with an increased risk of inherited arrhythmia, either LQTS or HCM, not previously genetic tested and who were consecutively referred or self-referred to genetic counselling between the years 2005-2007 in Bergen, Trondheim or Oslo and (b) control subjects based on calculations from the normative data from the general Norwegian population [21].

a) The arrhythmia risk patients

One hundred and seventy-three patients were asked to participate in the study. Of these 39 (22.5%) did not consent to participate and 7 (5.2%) did not return the questionnaire, leaving 127 (73.4%) patients included in the study.

b) The general population

SF-36 expected scores were calculated based on the normative data from the general Norwegian population aged 19-80 that were randomly drawn from the Norwegian Population Register (n = 3500). In total 2323 (67%) responded [21].

### Procedure

Participants filled in a questionnaire measuring health status and socio-demographic variables, whereas their clinical status was obtained from the medical records. Information about the study and a consent form was mailed to the patient together with the questionnaire 2-4 weeks before the genetic counselling. The participants received one reminder. The study was approved by the Regional committee for medical research ethics in Western Norway in September 2004.

### Questionnaire

Short Form-36 Health Survey (SF-36) is a self-report questionnaire that measures health status domains (0 = worst health state. 100 = best health state) on eight sub-scales; where physical functioning (PF), role limitation-physical (RP), bodily pain (BP) and general health (GH) are mainly considered physical health domains and vitality (VT), social functioning (SF), role limitation-emotional (RE) and mental health (MH) are considered mental health domains. The general health and vitality domains have both physical and mental aspects in their construct. An additional point reports changes in health over the last year. The questionnaire is generic and multidimensional and it is suitable for administration to large populations and to subgroups such as patients. Its purpose is to be a measure of health status or health outcome in cross-sectional and longitudinal studies. The SF-36 has been shown to be a reliable and valid measure across studies all over the world and the Norwegian version exhibits satisfactory psychometric properties [21-24].

### Socio-demographic variables and clinical status

The socio-demographic variables age, sex, and marital status, children, employment and education status, heart-disease in the family, family history of sudden death and/or genetic testing, and referral by physician/self referral through family member were obtained from the self-report questionnaire. The clinical status was collected from the patients' medical record and defined as having a family risk of arrhythmia or having a clinical diagnosis of either LQTS or HCM.

### Statistical analysis

Descriptive analyses were performed for socio-demographic variables, clinical status and the health status domains of SF-36. Descriptive statistics for SF-36 are given as mean, standard deviation (SD) and number of participants. SF-36 expected scores were calculated based on the normative data from the general Norwegian population aged 19-80 years that were randomly drawn from the Norwegian Population Register (n = 3500) [21]. SF-36 expected scores for each of the respondents were calculated for all health status domains, controlling for age and sex. Bivariate analyses were performed using paired samples t-tests [25] when comparing health status in the different groups with expected scores. Non-parametric tests were considered for analyzing the two smaller subgroups, but gave the same results with regard to statistical significance as the parametric tests. It was therefore decided to present the results with the parametric analysis, reporting mean differences and p-values.

When measuring the impact of clinical status on levels of health status, one-way analysis of variance (anova) with post hoc comparisons, using the Tukey method to correct for multiple testing was used.

Multiple linear regression analyses were performed on the total arrhythmia-risk group to assess the impact of the socio-demographic variables and clinical status on the different health domains of SF-36. Models were reported as unstandardised regression coefficients (β) with standard error, p-values and determination coefficients (*R*^2^), adjusted. From a fully adjusted model with all socio-demographic variables and clinical status entered, a backward stepwise elimination of predictors were performed to achieve a final model with variables fulfilling the inclusion criteria with p ≤ 0.05 and excluded with p ≥ 0.1 All other tests were two-tailed at the 5% significance level. Data were analyzed using SPSS version 15.0.

## Results

**Figure 1:**
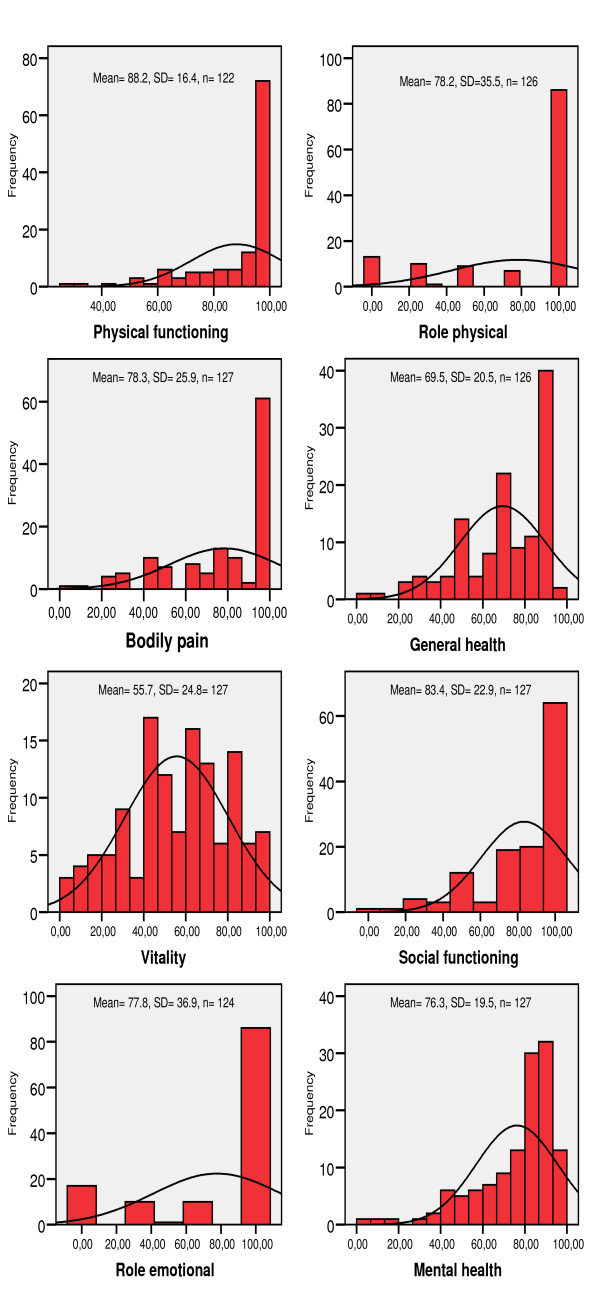
**Distribution of the SF-36 Health status scores**.

The socio-demographic and clinical status of the arrhythmia risk group is presented in table 1. The distributions of SF-36 health status scores for the total arrhythmia risk group are presented in figure 1, indicating some deviation from normal distribution.

**Table 1:** Socio-demographic variables and clinical status of the 127 arrhythmia risk patients coming to genetic counselling

Variable	Total n = 127	% 100
**Sex**		
Female	68	53.5
Male	59	46.5
**Age Groups**		
29 or less:	26	20.5
30-39:	20	15.7
40-49:	31	24.4
50-59:	27	21.3
60-69:	11	8.7
70 or more:	12	9.4
**Marital Status**		
Married/cohabitant	98	77.2
Single	17	13.4
Divorced/separated	7	5.5
Widow/widower	4	3.1
Missing	1	0.8
**Children**		
Have children	99	78.0
Missing	2	1.6
**Employment**	86	67.7
Missing	1	0.8
**Education Status**		
Primary school	26	20.5
High school	64	50.4
College/university	37	29.1
Missing		
**Heart-disease in family**		
Long QT syndrome (LQTS)	88	69.3
Hypertrophic cardiomyopathy (HCM)	39	30.7
**Sudden Death Occurred in Family**		
Sudden Death	57	44.9
Missing	25	19.7
**Genetic Testing Conducted in**		
**Family**		
Genetic Testing	77	60.6
Missing	14	11.0
**Referral**		
By physician	35	27.6
Self referred through family	90	70.9
Missing	2	1.6
**Clinical Status**		
Family risk	95	74.8
Clinical diagnosis of LQTS	12	9.4
Clinical diagnosis of HCM	20	15.7

### Comparisons with the expected scores of the general population

In table 2 the SF-36 health status scores among patients at risk of inherited arrhythmias compared to the expected scores of the general population is presented. After adjusting for age and sex the total arrhythmia risk group reported significant lower SF-36 health status score as compared to the general population scores for the domain of general health. When comparing the subgroups with the expected scores, the general health was still significant lower for the group of family risk and in the group of HCM. In addition the physical functioning, role physical, vitality and role emotional domains were reduced for the latter group. There were no significant differences between the patients with a clinical diagnose of LQTS and the expected scores, and the family risk group had better scores on the domains of physical health and bodily pain, than the general population.

**Table 2:** SF-36 Health status scores among patients at risk of inherited arrhythmias compared to the expected scores of the general population*

SF-36 Health status domains	Clinical status	n	Mean (SD)	**Mean diff**.	p-value
**Physical functioning**	Total sample	122	88.2(16.4)	1.1	0.389
	Family risk	91	90.6(14.2)	3.7	<0.001
	LQTS	12	93.3(14.2)	1.7	0.678
	HCM	19	73.2(19.5)	-11.7	0.021
**Role physical**	Total sample	126	78.2(35.5)	0.3	0.915
	Family risk	95	83.2(31.5)	5.1	0.095
	LQTS	12	83.3(34.3)	-0.6	0.956
	HCM	19	50.0(43.3)	-23.3	0.024
**Bodily pain**	Total sample	127	78.3(25.9)	3.4	0.138
	Family risk	95	82.2(23.0)	7.1	0.003
	LQTS	12	75.2(30.2)	-2.9	0.735
	HCM	20	62.0(30.8)	-10.9	0.127
**General health**	Total sample	126	69.5(20.5)	-7.3	<0.001
	Family risk	94	72.6(18.1)	-4.6	0.012
	LQTS	12	71.3(22.8)	-8.5	0.224
	HCM	20	54.1(23.9)	-19.1	0.004
**Vitality**	Total sample	127	55.7(24.8)	-4.3	0.530
	Family risk	95	58.3(24.7)	-1.3	0.602
	LQTS	12	53.9(29.5)	-6.2	0.488
	HCM	20	44.5(19.5)	-17.4	0.001
**Social functioning**	Total sample	127	83.4(22.9)	-2.3	0.245
	Family risk	95	85.4(20.4)	-0.0	0.986
	LQTS	12	83.3(29.4)	-3.4	0.693
	HCM	20	61.4(43.4)	-12.7	0.068
**Role emotional**	Total sample	124	77.8(36.9)	-4.0	0.217
	Family risk	93	80.5(34.6)	-1.2	0.734
	LQTS	12	83.3(38.9)	-0.3	0.976
	HCM	19	61.4(43.4)	-20.1	0.054
**Mental health**	Total sample	127	76.3(19.5)	-2.5	0.155
	Family risk	95	75.9(19.8)	-2.6	0.197
	LQTS	12	79.1(25.3)	0.8	0.912
	HCM	20	76.3(14.0)	-3.6	0.245

* Paired samples t-tests between the total sample, groups and expected scores of the general population reporting number of participants, means, standard deviations, mean differences and p-values.

### Comparisons between the groups according to clinical status

Table 3 report the impact of clinical status on levels of health status, as measured with the eight SF-36 health status domains. Patients with a clinical diagnosis of HCM had significantly lower scores than the patients with a clinical diagnosis of LQTS and the family risk group on all physical health status domains, with the exception of the domain of bodily pain where the mean difference between having a diagnosis of LQTS or HCM was non-significant. The most striking result was the difference on the role physical domain (50 vs. 83.3 and 83.2). There were no statistically significant differences in mental health status domains for the three groups.

**Table 3:** Differences between groups according to clinical status*

SF-36 Health status domains	Clinical status	*F*-test statistic (p-value)	**Mean diff**.	p-value
**Physical functioning**	Family risk-LQTS	*F*_2,121 _= 11.3	-2.7	0.831
	LQTS-HCM	(<0.001)	20.2	0.001
	Family risk-HCM		17.5	<0.001
**Role physical**	Family risk-LQTS	*F*_2,125 _= 7.8	-0.1	1.000
	LQTS-HCM	(0.001)	33.3	0.023
	Family risk-HCM		33.2	<0.001
**Bodily pain**	Family risk-LQTS	*F*_2,126 _= 5,5	7.0	0.632
	LQTS-HCM	(0.005)	13.2	0.324
	Family risk-HCM		20.2	0.004
**General health**	Family risk-LQTS	*F*_2,126 _= 7.5	1.3	0.975
	LQTS-HCM	(0.001)	17.3	0.044
	Family risk-HCM		18.6	0.001
**Vitality**	Family risk-LQTS	*F*_2,125 _= 2.7	4.4	0.824
	LQTS-HCM	(0.073)	9.4	0.547
	Family risk-HCM		13.8	0.060
**Social functioning**	Family risk-LQTS	*F*_2,126 _= 2.2	2.1	0.953
	LQTS-HCM	(0.117)	9.6	0.481
	Family risk-HCM		11.6	0.096
**Role emotional**	Family risk-LQTS	*F*_2,125 _= 2.3	-2.9	0.964
	LQTS-HCM	(0.104)	21.9	0.237
	Family risk-HCM		19.1	0.099
**Mental health**	Family risk-LQTS	*F*_2,126 _= 0.1	-3.1	0.860
	LQTS-HCM	(0.872)	2.8	0.920
	Family risk-HCM		-0.4	0.997

*ANOVA with Tukey correction for multiple post hoc tests for the health status domains.

### The relationship between socio-demographic variables and health status domains

In table 4 multiple regression analyses showed that socio-demographic variables and clinical status could explain considerable variability in the different health status domains, ranging from 30.2% to 51.8%. Of the physical health domains it was physical functioning that was best explained by the socio-demographic variables (51.8%), while of the mental domains it was social functioning (37.9%). Sex, age, employment and educational status, family history of heart disease or sudden death, referral by physician vs. family member, and clinical status showed significant relationships with different health status domains. Women scored lower than men on all scales for bodily pain, vitality, and mental health domains. Increasing age had a significant relationship to reduced physical function and role-physical. Being employed was related significantly to an increase of all health domains, except for the mental health domain, that was non-significant. Higher education status was significantly associated to less bodily pain, better general health, more vitality, higher degrees of social functioning and better mental health. A family history of HCM had a significant negative relationship with physical and general health. Referral to genetic counselling by a physician had a significant negative relationship to all the health domains. The clinical status of having the diagnosis of LQTS had a significant positive relationship with the mental health domain.

**Table 4:** Multiple linear regression analysis assessing the relationship between socio-demographic variables, clinical status and the SF-36 health status domains among the arrhythmia risk patients*

SF-36 Health status domains	Socio-demographic variables and clinical status	β	Standard error	p-value	***R***^2^
**Physical functioning**	Age	-0.3	0.08	<0.001	*0.518*
	Employment	12.7	2.64	<0.001	
	Heart disease in family	-8.2	2.86	0.005	
	Referral	-8.9	2.84	0.002	
**Role physical**	Age	-0.5	0.19	0.013	*0.390*
	Employment	27.8	6.55	<0.001	
	Genetic testing in family	7.1	4.19	0.095	
	Referral	-22.6	7.94	0.005	
**Bodily pain**	Sex	13.3	4.40	0.003	*0.326*
	Employment	13.4	4.89	0.008	
	Education status	6.8	3.15	0.032	
	Sudden death in the family	8.7	4.45	0.054	
	Referral	-22.7	4.98	<0.001	
**General health**	Employment	17.5	3.77	<0.001	*0.391*
	Education status	4.4	2.43	0.073	
	Heart disease in the family	-8.0	4.05	0.052	
	Referral	-14.8	4.10	0.001	
**Vitality**	Sex	10.0	4.44	0.027	*0.302*
	Employment	11.8	4.95	0.019	
	Education status	11.0	3.19	0.001	
	Referral	-18.2	5.05	0.001	
**Social functioning**	Employment	12.1	4.22	0.005	*0.379*
	Education status	10.6	2.72	<0.001	
	Referral	-20.3	4.30	<0.001	
**Role emotional**	Employment	33.3	7.07	<0.001	*0.320*
	Referral	-31.2	7.39	<0.001	
**Mental health**	Sex	8.4	3.52	0.019	*0.316*
	Children	-7.5	4.49	0.097	
	Education status	11.5	2.42	<0.001	
	Referral	-20.9	3.47	0.001	
	Clinical status	7.2	5.84	0.040	

*Model developed from backward stepwise selection of all socio-demographic and clinical predictors from the fully adjusted model fulfilling the inclusion criteria with p = 0.05 and exclusion criteria with p > 0.1 reported with unstandardised coefficients (β), standard error, p-values (p) and Adjusted R Square (*R*^2^). Coding of the independent variables: sex; female = 0, male = 1, children; no = 0, yes = 1, employment; no = 0, yes = 1, education status; primary school = 1, high school = 2, college/university = 3, heart disease in family; LQTS = 1, HCM = 2, sudden death in family; no = 0, yes = 1, genetic testing in family; no = 0, yes = 1, referral; by family member = 0, by physician = 1, clinical status; family risk = 0, LQTS = 1, HCM = 2. Age was treated as continuous. The dependent variables: The SF-36 health status domains were treated as continuous (0-100).

## Discussion

The present study demonstrates reduced health status in the SF-36 domain of general health in the arrhythmia-risk patients. The general health domain was reported significantly lower than the general population with the largest mean difference. In general, employment, higher education and being referred to genetic counselling through a family member were associated with better scores on the health status domains.

The general health domain deals with a personal evaluation of both physical and mental health, including current health, health outlook, and resistance to illness. In the present study, the general health domain was reduced among patients at genetic risk of arrhythmia, even if the other physical health domains were indifferent or even better than the expected scores from the general population. The mental health domains were less than the reference scores, but none of them reached statistical significance. Even if the finding may seem inconsistent, considering what these patients are up against, it might not be surprising. Living with and having knowledge about genetic risk of sudden death in the family can initiate genetic testing and health preventive behaviours, but one can also imagine that it will have an effect on how you perceive your health and health outlook.

According to a psychological framework for analysing risk of disease, the risk can be defined in terms of *probability and effect *[26]. The probability part of the risk concept is the likelihood of some specific negative event will occur as a result of the possible genetic vulnerability. For most patients in our study there is a high probability for heart disease, since the genetic vulnerability for heart disease is already known among first degree family members. The probability for having inherited mutation associated with heart disease is 50% (autosomal dominant inheritance), although, the penetrance for actually developing the disease as well as the intra-familial expression of the disease are varying. The effect side of the risk concept is the severity or consequence of the event. In a proportion of cases the event is very serious and even fatal in this patient population. With this in mind it can be the patients' relation to living with this risk that explain why they rate their general health poorly. It has been shown that uncertainty about survival can decrease the perception of general health [8,9], and Loge et al discusses their similar finding among Hodgkin's disease survivors, that it may reflect having lived with a potentially fatal disease [27]. Our participants are currently living with the risk of a potentially fatal disease, but this does not seem to influence the other health domains.

In other patient groups coming to genetic counselling, there have also been similar findings. In a study of women with high risk for breast cancer due to family history, subjects reported significant lower levels of health status for domains related to mental health and for general health compared to normative data, but similar levels on domains related to physical health [28].

There were also differences in health status between the subgroups and the expected scores of the general population, and differences between the groups according to clinical status. Patients with HCM had significantly reduced scores on physical health status domains compared to both the expected scores of the general population, the patients with LQTS and the family risk group, and reduced scores of the domains of vitality and role emotional compared to the general population. This is comparable with results from a study of health related quality of life and psychological wellbeing in patients with HCM, where the analysis of the SF-36 indicated limitations in both the physical and mental health domains [17]. In our study the LQTS group also demonstrated lower scores on most of the health domains but none were significant. This can be due to the nature of the diseases, where HCM will have more manifestations of physical symptoms that are more prone to affect especially physical health domains, whereas having a clinical diagnosis of LQTS or family risk may cause more worries and distress because there might be more uncertainties around symptoms, diagnosis and management of the disease, potentially affecting more psychological measures as previous research have shown [13,15,29-31].

In the present study, women scored significantly lower than men on the domains of bodily pain, vitality and mental health, domains that primarily measure well-being, whereas not for the domains that measures disability, such as physical functioning, role-physical, social functioning and role-emotional. One explanation of this might be that it is different for men and women to live with the risk of uncertainty. It has been previously shown that impact of uncertainty associated with children's chronic health condition can have a relationship with mothers mental and physical health, while uncertainty does not affect fathers' health in the same way[12]. In the normative data of the general Norwegian population all of the physical health status domains had a strong relationship with age, and in three out of four physical health domains women scored lower than men [21].

Being employed had a positive relationship to both physical and mental health domains in our study, which is in line with epidemiological research conducted in Norway that found better perceived health status in employed, as compared to unemployed and disability benefit recipients [32]. Lower education status predicted also lower health status as reported from the normative data of the Norwegian general population [21].

In a study of the psychological impact of risk for long QT syndrome, parents of carrier children reported high levels of distress. The distress was predicted by lower education status along with previous history of distress in person, knowledge of the disease for a longer time period, sudden death in the family, and unsatisfied with the disease-information [15]. In the present study reduced scores on the mental health domain is best predicted by being female, having children, lower education status and referral by physician, while experience of sudden death in the family did not have a significant relationship to mental health.

### Implications for genetic counselling practice

The main aim of the genetic counselling is to help people to understand and adapt to the medical, psychological, and familial implications of genetic contributions to disease. Therefore, it is important to have knowledge about the health status of these individuals coming to genetic counselling in order to be able to interpret, educate and counsel [33].

In the present study it was found that the socio-demographic variable influencing the health domains the most was how the patient was referred to genetic counselling. Being referred by a physician consistently showed a negative relationship to all health status domains. Other research has revealed that the patients experience a lack of knowledge and understanding concerning inherited arrhythmias among health-care providers [15,17,18,34], which previously have been described to have the potential of creating uncertainty, lead to misinformation and wrong treatment advice [16]. Being referred by a physician might influence the patient's perception and understanding of the content of information that is received in this setting compared to when patients are self-referred through a family member. Although it might also be that the patients rate their condition more seriously if the health care provider refers them to genetic counselling, expecting it is more serious then, or that the patients referred by the physician do have actual symptoms of disease. Regardless, being referred to genetic counselling will provide the possibility of getting the information necessary to be able to adapt to and understand the situation, which according to previous research [17] can be key predictors to an improved general health status in inherited arrhythmias.

### Limitations

The design of this study shares the limitations that all cross-sectional designs have regarding control, causality and generalisability. Our sample size was relatively small; however the data was collected at three different hospitals in three different health regions of Norway to reduce possible influence of community characteristics.

An important issue in discussing the findings in the present study are whether the research sample is representative of a greater population and what kind of biases might influence the results. Ideally we would like to generalize the finding in our study to all subjects undergoing genetic counselling for LQTS and HCM. The rate of decliners in the study was fairly high (26.6%). The Regional Committee for Medical and Health Research Ethics did not permit publication of data related to information for individuals who did not consent for research. Therefore, it was not possible to compare respondents to non-respondents. We cannot rule out the possibility that the decliners are different than the respondents. More health problems among non-responders [35], could be an alternative explanation for the somewhat better health scores in the present study, than what could have been expected.

The arrhythmia risk groups were different from each other with the distribution of some of the socio-demographic variables, but even if this mostly was controlled for in the analysis, there can be other variables that influence also. In the genetic counselling setting it is however interesting to analyse the groups together since it is not so much the clinical manifestations that are central to the research questions, but more the attempt to elucidate a major unmeasured component of overall health risk, the extent to which knowledge gained about the potential harm associated with the risk of disease affects the outcome of the subjective health status of the patients coming to genetic counselling. We were able in this study to explain considerable variance in the dependent variables with the socio-demographic variables and clinical status. However some variance in health status is yet to be explained, such as the influence of psychosocial variables.

## Conclusions

In conclusion, living with genetic risk of arrhythmia and possible sudden death is most likely related to health status vulnerability. In general, persons at genetic risk of arrhythmia perceive their current health; health outlook and resistance to illness to be lower than the general population, and for persons with HCM, physical health and emotional problems can in addition limit and interfere with work or daily activities. Lower health status reported on general health suggests that the arrhythmia-risk patients indeed is a special patient-group, a finding adding to the knowledge that this patient group can benefit particularly from genetic counselling. Prospective studies might give more insights to the effect of genetic counselling.

## Competing interests

The authors declare that they have no competing interests.

## Authors' contributions

The authors' contributions to the work were as follows: conception and design (AH, BR, CB, NØ); analysis and interpretation of data (all authors); drafting the manuscript (AH); revising the manuscript critically for important intellectual content (all authors); and final approval of the manuscript submitted (all authors).

## Pre-publication history

The pre-publication history for this paper can be accessed here:

http://www.biomedcentral.com/1471-2350/11/27/prepub
